# A cross-sectional survey of medical and other groups’ awareness, perceptions, and willingness to use e-cigarettes during the COVID-19 pandemic

**DOI:** 10.3389/fpubh.2023.1323804

**Published:** 2024-01-08

**Authors:** Ruihang Deng, Chuang Yang, Yifang Yuan, Lifang Liang, Xin Yang, Xinyu Wang, Jiao Tian, Yuxin Zhang, Xuekai Wu, Haiyun Dai

**Affiliations:** ^1^First Clinical Medical College, Chongqing Medical University, Chongqing, China; ^2^Department of Respiratory and Critical Care Medicine, The First Affiliated Hospital of Chongqing Medical University, Chongqing, China

**Keywords:** COVID-19 pandemic, e-cigarettes, medical group, medical students, smoking cessation

## Abstract

**Background:**

In China, people’s perceptions towards electronic cigarettes during Corona Virus Disease 2019 (COVID-19) pandemic compared with pre-pandemic conditions have not been explored. Exploring the perceptions of medical workers regarding e-cigarettes is crucial, as they serve as a trusted source of information and providers of smoking cessation counselling for smokers. This cross-sectional study was designed to explore the awareness and perceptions of e-cigarettes among Chinese medical and other groups in the context of the COVID-19 pandemic.

**Methods:**

A cross-sectional survey was performed using an online, anonymous, and self-administered questionnaire. The questionnaire contained sections for collecting participants’ general information and Likert scale questions regarding smoking status, perceptions of e-smoking, attitude, and willingness to use e-cigarettes. The respondents included medical students, clinical doctors, and other occupations. Data analysis was performed using tools such as descriptive analysis, binary logistic regression, and multivariate regression.

**Results:**

A total of 952 people completed the questionnaire, and 96.54% of them reported to have heard about e-cigarettes. The most common source of information about e-cigarettes was advertising. Notably, 28 of the 116 smokers reported that they had used e-cigarettes. Independent-samples *T*-tests results showed that medical groups believed e-cigarettes contained tar (*p* = 0.03). Most of the medical and non-medical participants maintained neutral attitudes towards e-cigarette policies (38.3%) and prices (49.2%) but their views were significantly different (*p* < 0.001). Multivariate logistic regression indicated that highly educated people had higher knowledge about e-cigarettes relative to those with lower education (undergraduate, OR = 1.848, 95CI% = 1.305–2.616, *p* = 0.001; master’s degree or doctoral degree, OR = 1.920, 95CI% = 1.230–2.997, *p* = 0.004). The medical group used fewer e-cigarettes compared to non-medical group (OR = 1.866, 95CI% = 1.185–2.938, *p* = 0.007), the non-traditional cigarette users showed lower utilization compared to traditional cigarette users (18–40, OR = 4.797, 95CI% = 0.930–24.744, *p* = 0.061; > 40, OR = 9.794, 95CI% = 1.683–56.989, *p* = 0.011) and the older adult used fewer than the young (18–40, OR = 4.797, 95CI% = 0.930–24.744, *p* = 0.061; > 40, OR = 9.794, 95CI% = 1.683–56.989, *p* = 0.011).

**Conclusion:**

This study found that individuals tend to hold negative attitudes towards the awareness, perceptions, and willingness to use e-cigarettes. Medical groups are less likely to use e-cigarettes, but misperceptions are still prevalent among them. This calls for additional training for such medical personnel to improve their capacity to provide necessary counselling to smokers. E-cigarettes advertisements were the main source of information for young individuals to learn about e-cigarettes, and hence measures should be taken to restrict exposure of young individuals to e-cigarettes.

## Introduction

Electronic cigarettes (e-cigarettes) are made from batteries, atomizers, and combustible liquid (1). E-cigarettes, designed to mimic the appearance and smoking experience of traditional cigarettes without burning tobacco, operate by directly heating a nicotine-containing liquid through battery activation, producing an aerosol for inhalation (2). They are an alternative product to conventional cigarettes and its usage has been increasing in recent years (3). Corona Virus Disease 2019 (COVID-19) is an acute respiratory infection caused by 2019-nCoV infection (later was named SARS-CoV-2 by ICTV on February 11, 2020) (4). Since the World Health Organization declared COVID-19 a global pandemic on March 11th, 2020, there have been numerous changes in various aspects of people’s lives. Consequently, there have been changes in behaviors and perceptions towards e-cigarettes among their users, such as smoking cessation or increased usage due to elevated stress levels (5, 6). Although much research has focused on e-cigarette users, the perception and knowledge of e-cigarettes among non-e-cigarette users during the pandemic have not been explored sufficiently. This calls for further understanding of their perception and knowledge as it is crucial to tobacco control and formulating related policies.

The knowledge levels, reasons for use and access to e-cigarettes have been investigated previously (7–10). Of note, the attitudes towards e-cigarettes among various groups of people during the COVID-19 pandemic have changed in comparison to pre-pandemic times. This investigation can contribute to the refinement and customization of regulatory measures regarding e-cigarettes in the context of the pandemic. To a certain extent, the attitudes, knowledge, and inclination towards tobacco product use within the medical community provide insights into the practical effectiveness of tobacco control in our nation. Studies have shown that doctors provide fewer interventions for e-cigarette users and do not provide sufficient attention to them (11). For medical students, tobacco education is not well accepted due to inadequate training programs in tobacco cessation counselling in medical schools, and the high prevalence of misconceptions about e-cigarettes among them (12–14). Currently, relevant surveys exploring perceptions towards e-cigarettes in the medical group are lacking. During the pandemic, people’s health awareness has been greatly improved and many e-cigarette users have chosen to quit smoking in such circumstances (5, 15). Given that medical professionals are often viewed as authoritative sources of information for smokers, it is important to delve into their perceptions of e-cigarettes.

At present, the Chinese government has implemented certain policies and regulations on e-cigarettes such as regulating the production and sale of e-cigarettes and establishing guidelines for advertising restrictions. However, it is worth noting that the e-cigarette market in China is still evolving, and the effectiveness and enforcement of these policies may vary across different regions. Moreover, there are currently no detailed regulations pertaining the use of e-cigarettes. Against this backdrop, we conducted a cross-sectional survey to evaluate the awareness and receptivity towards e-cigarettes among various groups in China, particularly within the context of the COVID-19 pandemic. Our aim was to gain insights into the challenges and opportunities associated with e-cigarette use in China and uncover potential strategies and policies that could effectively curb e-cigarette use and promote smoking cessation.

## Methods

### Setting and participants

This was a cross-sectional survey using online questionnaires, conducted from November 2021 to January 2022. The survey was administered to medical students (Undergraduate and graduate), practicing doctors from Chongqing Medical University, and other non-medical professionals. At Chongqing Medical University, all students from every grade and doctors from every department were members of their respective communication groups. To ensure effective distribution of the questionnaire, we reached out to the managers of these groups and requested their assistance in circulating the online questionnaire link among the group members. Participation in the study was voluntary, and potential participants were required to complete all sections of the questionnaire before submission, ensuring a 100% completion rate. To prevent duplicate responses, each individual was restricted to filling out the online questionnaire only once.

The sample size for this study was determined using the Rao soft samplesize calculator.^1^ With a confidence level of 95%, a margin of error of 5%, and assuming a response distribution of 50%, the recommended sample size was determined to be 377. After exclusion of three age outliers, a total of 952 participants were enrolled. Each participant provided an informed consent form to be enrolled in the study after being informed of the purpose of the study.

### Questionnaire design

The survey used a self-administered scale modelled on the World Health Organization’s Global Adult Tobacco Survey (GATS) and the scale of adolescents’ use and attitude towards e-cigarettes (16, 17). The questions were formulated to suit the purpose of the survey. After review by experts, pre-surveys and semi-structured interviews were conducted before the official release of the final questionnaire (*n* = 96). The reliability and validity of the scale were tested using the pre-survey data, and the final questionnaire was developed after several screening methods (Attached document 1). The questionnaire (Attached document 2) contained questions designed to obtain the following information: participants’ general information (4 questions), smoking status (9 questions), perception of e-smoking (7 questions), attitudes (2 questions) and willingness to use (4 questions), totaling 26 questions.

Participants’ general information (question 1 to 4): The demographic section asked participants about their age, gender, occupation and education background.

Smoking status (question 5 to 13): Most of the questions in this part of the questionnaire were based on GATS. Questions 5 to 8 asked participants about their smoking status and reasons for smoking, and questions 9 to 13 asked participants about the ways they know about e-cigarettes and their use of e-cigarettes, as well as other questions related to e-cigarettes.

Perception of e-smoking (question 14 to 19): The section of “perception” investigates the level of agreement among participants regarding the viewpoints presented in the survey questionnaire. The questions are related to e-cigarette knowledge and the correct answers to all the questions are contrary to the description of the questions, aiming to evaluate their especially among medical professionals level of reserve of knowledge on e-cigarettes to provide tobacco cessation counselling.

Attitude (question 20 to 21): The following questions in Likert scale are subjective and there is no correct answer, which mainly compared the participants’ different attitudes towards e-cigarettes, traditional cigarettes and electronic cigarette related policies.

Willingness (question 22 to 26): Similar to the questions in the “Attitudes” section, these closed-end questions, with no correct answer, were designed to assess the likelihood of respondents using e-cigarettes due to the influence of surrounding smokers.

Most of the questions regarding smoking were answered with closed-ended responses. The Likert scale was applied in questions 14 to 26 of the questionnaire. The score indicated the degree of concurrence with the question, ranging from 1 for “strongly disagree” to 5 for “strongly agree.” The average score of the participants for each item on the scale was used to represent the degree of agreement.

### Data analysis

The data from the online survey was first exported to EXCEL and then transferred to IBM SPSS software (version 26.0.0.0) for statistical analysis. To evaluate the internal consistency of the items within the principal components, Cronbach’s alpha was employed (18). The questionnaire’s structural validity was investigated based on the exploratory factor analysis by calculating Kaiser-Meyer-Olkin (KMO) value and Bartlett sphericity test *p*-value (19), which indicated the feasibility of factor analysis. The rotated component matrix was derived using the maximum variance method. Descriptive analysis was conducted on fundamental demographic information and smoking status. Basic demographic factors were incorporated into the multivariate regression analysis. The Mann–Whitney Test was utilized to compare the scores of different occupations on Likert scale questions. Binary logistic regression was employed to identify the independent factors affecting individuals’ knowledge, attitude, and willingness to use e-cigarettes. Odds ratios (OR) and 95% confidence intervals (95%CI) were calculated at a significance level of *p* = 0.05.

### Ethical considerations

This study did not include any human clinical trials or animal experiments. An anonymous online questionnaire survey was employed which did not involve ethical issues related to human trials. Based on the guidelines of relevant institutions and national laws and regulations, there was no need for approval from the ethics committee.

## Result

### Construct validity analysis

After conducting exploratory factor analysis, the questionnaire’s KMO value was 0.754 with X^2^ = 637.8 of the Bartlett sphericity test. A *p* < 0.001 was obtained which indicated its suitability for factor analysis. Through the maximum variation method, characteristic root factors greater than 1 were identified as common factors. From the 16 scale questions, a total of four common factors were extracted, contributing to a cumulative variance of 64.533%. This indicates that these four factors can elucidate 64.533% of the questionnaire’s content. Therefore, it can be inferred that the four identified common factors effectively encapsulate and elucidate the information from the original variables.

### Reliability analysis

The Cronbach’s alpha of each dimension in the scale ranged from 0.659 to 0.856 and the Cronbach’s alpha of the total scale was 0.765, suggesting that the scale had good reliability and high internal consistency across the 16 scale questions.

### Data results

A total of 952 participants were enrolled in this study, with the majority (86.60%) aged between 18 and 40. More than half (59.48%) of the participants were female. Moreover, 52.57% of the participants’ occupations were related to medicine. The majority of the participants were undergraduates (56.34%). The basic information of the participants is presented in Table 1.

**Table 1:** Demographics and characteristics of participants.

Characteristic	*N* (%)
Age	0–17 (1.60%)
	18–40 (86.60%)
	>40 (11.90%)
Smoking rates	0–17 (6.7%, 1/15)
	18–40 (11.3%, 93/824)
	>40 (20.4%, 23/113)
Gender	Female 568 (59.48%)
	Male 387 (40.52%)
Profession	Non-medical related 453 (47.43%)
	Medical-related 502 (52.57%)
Education background	High school and below 151 (15.81%)
	Junior college 77 (8.06%)
	Bachelor’s degree 538 (56.34%)
	Master’s degree 152 (15.92%)
	Doctor’s degree 37 (3.87%)

Table 2 displays the smoking habits of the participants. Only 7.02% of the participants were current daily smokers while non-daily smokers accounted for 5.34% of the participants. Within this group, a follow-up question indicated that 37.25% had been daily smokers in the past, whereas 58.82% denied it. The remaining 87.64% of non-smoking participants were asked the third question, which revealed that 95.22% of participants did not have a history of smoking. For smokers, there were several reasons for starting smoking, which includes the influence of smoking around 49 (41.53%), feeling bored and to killing time 39 (33.05%), social needs 48 (40.68%), need to try something new 22 (18.64%), release the pressure 69 (58.47%), and smoking for concentration 5 (4.24%).

**Table 2:** Participants’ smoking habits.

Smoking habits of participants	*N* (%)
Are you smoking now?	Everyday 67 (7.02%)
	Yes, but not every day 51 (5.34%)
	No 837 (87.64%)
Have you ever smoked every day before?	Yes 19 (37.25%)
	No 30 (58.82%)
	Unclear 2 (3.92%)
Have you ever smoked before?	Everyday 9 (1.08%)
	Yes, but not every day 31 (3.7%)
	No 797 (95.22%)
What caused you to start smoking?	The influence of smokers around 49 (41.53%)
	Feel bored and to kill time 39 (33.05%)
	Social needs 48 (40.68%)
	Try something new 22 (18.64%)
	Release the pressure 69 (58.47%)
	Other reason 5 (4.24%)

**Figure 1:**
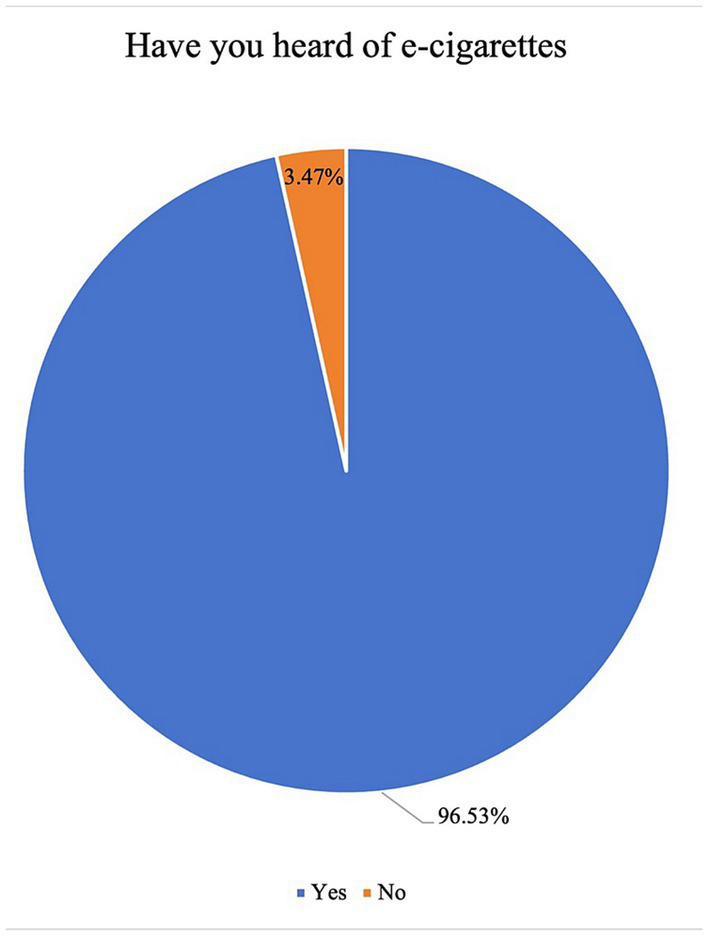
Awareness rate of electronic cigarettes.

**Figure 2:**
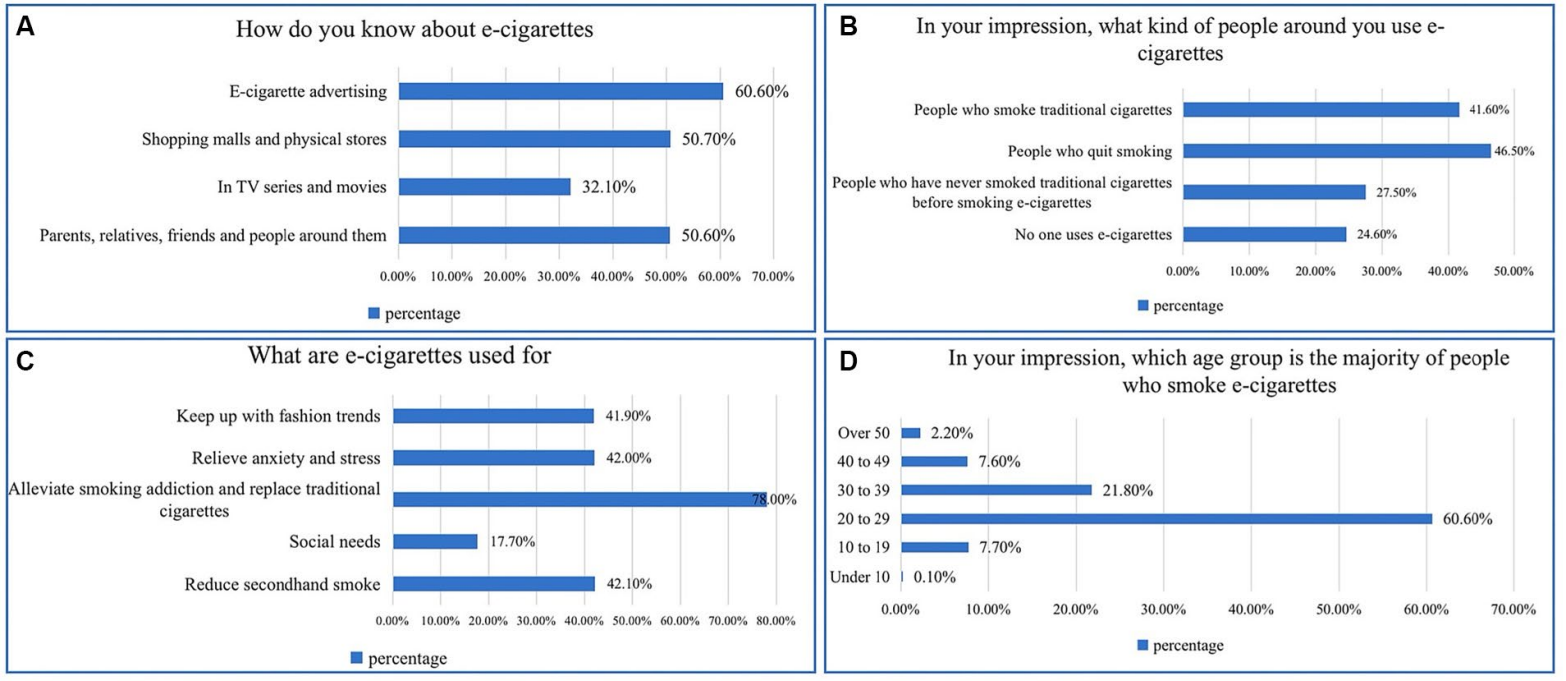
Participants’ perceptions of e-cigarettes. **(A)** Information sources for e-cigarettes. **(B)** E-cigarette users. **(C)** The purpose of using e-cigarettes. **(D)** Common age groups for smoking e-cigarettes.

Of note, 96.53% of the participants reported having heard of e-cigarettes, while the rest 3.47% of the participants did not complete the questionnaire (Figure 1). The remaining 919 participants were posed with four specific questions. Results revealed that the primary source of information about e-cigarettes for 60.60% of participants was e-cigarette advertising (Figure 2A). It was observed that individuals who had quit smoking, as well as those who still smoked conventional cigarettes, were more inclined to use e-cigarettes (Figure 2B). Additionally, a significant majority (78.00%) believed that e-cigarettes were used to combat smoking addiction and serve as an alternative to traditional cigarettes (Figure 2C). The age group between 20 and 29 was identified as the most likely to use e-cigarettes (Figure 2D).

Table 3 shows the participants’ agreement levels with specific viewpoints. The analysis revealed that 35.6% of the participants strongly disagreed that e-cigarettes are healthier than traditional cigarettes. Almost no one thought that e-cigarettes do not contain tar (2.0%). Furthermore, 38.5% strongly agreed that e-cigarettes would not be addictive. 32.6% of participants were neutral about e-cigarettes as a smoking cessation alternative to traditional cigarettes. It was further showed that 56.5% of participants had an unfavorable opinion about the negative effects of second-hand smoke from e-cigarettes. A notable 42.9% of participants strongly disagreed with the notion of using e-cigarettes indoors. Additionally, there was a lack of strong inclination towards using e-cigarettes, with 41.9% expressing neutrality in response to the statement, “I’d rather use e-cigarettes than traditional cigarettes.” Part of them mainly showed neutral attitude towards e-cigarette policy (38.3%) and price (49.2%). But most participants showed negative attitudes concerning e-cigarette use. The majority (81.1%) strongly indicated that they would not use e-cigarettes in the next 12 months and strongly disagreed with ever having used e-cigarettes. In addition, 75.6% of the respondents strongly indicated that the use of e-cigarettes around them would not make them want to smoke e-cigarettes.

**Table 3:** Participants’ agreement levels of the questions.

		Strongly disagree	Disagree	Neutral	Agree	Strongly agree	*p* value
Perception	E-cigarettes are healthier than traditional cigarettes	327 (35.6%)	233 (25.4%)	316 (34.4%)	39 (4.2%)	4 (0.4%)	0.108
	E-cigarettes contain tar	18 (2.0%)	115 (12.5%)	326 (35.5%)	203 (22.1%)	257 (28.0%)	0.030
	E-cigarettes are not addictive	354 (38.5%)	251 (27.3%)	254 (27.6%)	44 (4.8%)	16 (1.7%)	0.018
	E-cigarettes can replace traditional cigarettes and help quit smoking	283 (30.8%)	219 (23.8%)	300 (32.6%)	108 (11.8%)	9 (1.0%)	0.055
	E-cigarettes have no harm from second-hand smoke	309 (33.6%)	210 (22.9%)	258 (28.1%)	124 (13.5%)	18 (2.0%)	0.072
	E-cigarette use is permitted in homes or indoor places such as offices or shopping malls	394 (42.9%)	232 (25.2%)	220 (23.9%)	62 (6.7%)	11 (1.2%)	0.042
	I’d rather use e-cigarettes than traditional cigarettes	238 (25.9%)	87 (9.5%)	385 (41.9%)	180 (19.6%)	29 (3.2%)	0.887
Attitude	Raising the legal age for e-cigarettes is a bad thing	123 (13.4%)	176 (19.2%)	352 (38.3%)	100 (10.9%)	168 (18.3%)	0.909
	The price of e-cigarettes is too high	10 (1.1%)	52 (5.7%)	452 (49.2%)	220 (23.9%)	185 (20.1%)	0.133
Willingness	Some of my family, friends, and people around me smoke e-cigarettes	262 (28.5%)	144 (15.7%)	198 (21.5%)	248 (27.0%)	67 (7.3%)	<0.001
	In the next 12 months, I may use e-cigarettes	745 (81.1%)	50 (5.4%)	95 (10.3%)	15 (1.6%)	14 (1.5%)	<0.001
	I have used electronic cigarettes	754 (81.1%)	29 (5.4%)	72 (7.8%)	27 (2.9%)	37 (4.0%)	<0.001
	People around me who smoke e-cigarettes will make me want to smoke e-cigarettes	695 (75.6%)	55 (6.0%)	125 (13.6%)	38 (4.1%)	6 (0.7%)	<0.001

*p*-value analyzes the differences between different Professions in each item by Mann–Whitney Test.

Table 4 analyzes the differences between medical and non-medical subjects in each item through Mann–Whitney test. There was a statistically significant difference in their perceptions towards the questions that e-cigarettes contain tar and that e-cigarettes are not addictive. Medical and non-medical participants revealed no significant difference in attitudes towards e-cigarettes (*p* > 0.05), while the usage of e-cigarettes (*p* < 0.001) varied significantly.

**Table 4:** The scores of awareness, attitude, and willingness to use e-cigarettes in different occupations.

		Medical*	Non-medical*	*p* value
Perception	E-cigarettes are healthier than traditional cigarettes	2.04	2.14	0.108
	E-cigarettes contain tar	2.31	2.46	0.030
	E-cigarettes are not addictive	1.96	2.14	0.018
	E-cigarettes can replace traditional cigarettes and help quit smoking	2.21	2.37	0.055
	E-cigarettes have no harm from second-hand smoke	2.21	2.34	0.072
	E-cigarette use is permitted in homes or indoor places such as offices or shopping malls	1.90	2.06	0.042
	I’d rather use e-cigarettes than traditional cigarettes	2.63	2.63	0.887
Attitude	Raising the legal age for e-cigarettes is a bad thing	2.98	2.95	0.909
	The price of e-cigarettes is too high	2.39	2.46	0.133
Willingness	Some of my family, friends, and people around me smoke e-cigarettes	2.52	2.83	<0.001
	In the next 12 months, I may use e-cigarettes	1.25	1.52	<0.001
	I have used electronic cigarettes	1.28	1.61	<0.001
	People around me who smoke e-cigarettes will make me want to smoke e-cigarettes	1.36	1.63	<0.001

*Indicates that the data is represented by the average score of different occupations. *p*-value analyzes the differences between different professions in each item by Mann–Whitney Test.

**Figure 3:**
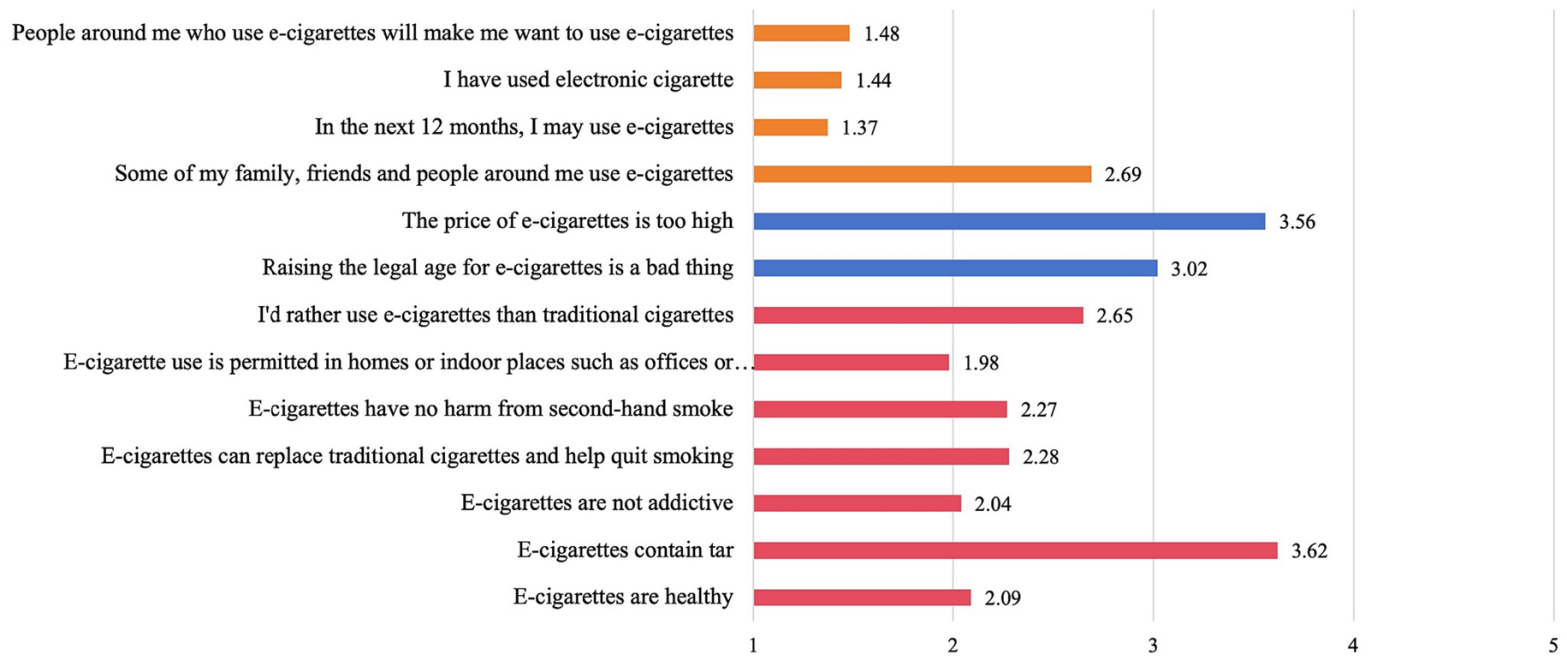
Mean of scale questionnaire.

The analysis revealed that participants tended to hold negative attitudes towards the cognitive and behavioral problems of e-cigarettes. Participants displayed a preference for e-cigarettes over traditional cigarettes (mean = 2.65). There was a consensus that e-cigarettes contain tar (mean = 3.62). Additionally, participants expressed a positive attitude towards the higher price of e-cigarettes (mean = 3.56). The overall score for e-cigarette use was low, indicating a minimal likelihood of using e-cigarettes in the next 12 months (mean = 1.37). Moreover, participants reported low personal use of e-cigarettes (mean = 1.44), and a limited influence from those around them who use e-cigarettes (mean = 1.48). That is, in the next 12 months, I may use e-cigarettes (mean = 1.37), I have used e-cigarettes (mean = 1.44), and people around me who use e-cigarettes will make me have the impulse to use e-cigarettes (mean = 1.48) (Figure 3).

Amount the factors found to be predictors of people’s perception, attitude, and willingness to use e-cigarettes for screening included age, gender, occupation, education background, and smoking status. Multivariate regression analysis showed that (Table 5) education background influences e-cigarette perception; occupation, smoking status and age were independent risk factors influencing the willingness to use e-cigarettes. Highly educated people tended to have higher cognitive level towards e-cigarettes compared with participants with lower education (undergraduate, OR = 1.848, 95CI% = 1.305–2.616, *p* = 0.001; master’s degree or doctoral degree, OR = 1.920, 95CI% = 1.230–2.997, *p* = 0.004). The medical group used fewer e-cigarettes relative to the non-medical group (OR = 1.866, 95CI% = 1.185–2.938, *p* = 0.007), the non-traditional cigarette users used fewer than traditional cigarette users (18–40, OR = 4.797, 95CI% = 0.930–24.744, *p* = 0.061; > 40, OR = 9.794, 95CI% = 1.683–56.989, *p* = 0.011) and the older adult used fewer than the young (18–40, OR = 4.797, 95CI% = 0.930–24.744, *p* = 0.061; > 40, OR = 9.794, 95CI% = 1.683–56.989, *p* = 0.011).

**Table 5:** Predictors of perception, attitude, and willingness to use e-cigarettes.

	Factor	OR(95%CI)	*p*-value
Perception	Educational background	High school and below or junior college	Ref
		Undergraduate 1.848 (1.305, 2.616)	0.001^a^
		Postgraduate 1.920 (1.230, 2.997)	0.004^a^
Willingness to use	Occupation	Non-medical profession	Ref
		Medical profession 1.866 (1.185, 2.938)	0.007^b^
	Smoking status	Smoking traditional cigarettes	Ref
		Not smoking traditional cigarettes 12.182 (7.613, 19.495)	*p* < 0.001^b^
	Age range	<18	Ref
		18–40 4.797 (0.930,24.744)	0.061^a^
		>40 9.794 (1.683,56.989)	0.011^a^

a Analyze group differences of perception for e-cigarettes on different educational background and refer to the Kruskal-Wallis Test.

b Analyze group differences of willingness to use e-cigarettes on different occupation, smoking status and age range with the Mann–Whitney Test.

## Discussion

To the best of our knowledge, this is the pioneer investigation into disparities in perceptions, attitudes and willingness to use e-cigarettes in China in the wake of the COVID-19 pandemic. Furthermore, it is the first report to compare perceptions between medical and non-medical groups in China. The survey comprised a substantial sample (*n* = 952), approximately half of which were current medical students as well as working physicians. The data indicates that individuals over 40 years of age have the highest prevalence of tobacco use. The 18–40 age group also exhibits a significant rate of tobacco use, suggesting that it is prevalent among both younger and middle-aged demographics. More than half of the participants reported that the reason for starting smoking was to relieve stress. In the study by Bennett et al. (6), it was found that the main reason for increased e-cigarette use among young adults during the COVID-19 pandemic was stress management. This suggests that effectively reducing stress and anxiety levels could play a pivotal role in helping individuals reduce their reliance on e-cigarettes and facilitate smoking cessation, especially during and after the COVID-19 period. In our study, 60.60% of respondents reported obtaining information about e-cigarettes through advertisements, and it was observed that young individuals are among the high-frequency users. Elsewhere, Gentzke et al. (20), investigated middle and high school students during the pandemic. They found that 75.7% of students exposed to the Internet, streaming media, convenience stores and other potential sources reported having received product promotion or advertising for tobacco products. Moreover, among students using social media, 73.5% reported receiving e-cigarettes related contents. This phenomenon may be linked to the heightened online presence and exposure of young individuals. Online media tends to showcase e-cigarettes in a more appealing light, making them more susceptible to marketing promotions. It’s worth noting, as pointed out by Brożek et al. (21), that some media advertisements, driven by profit motives, can be misleading by portraying e-cigarettes as safer alternatives to tobacco and effective aids for smoking cessation (which is not supported by evidence). Hence, it is imperative to implement stricter regulations on e-cigarette advertising, with a particular focus on monitoring online media discussions related to e-cigarettes. Several studies have reported the COVID-19 pandemic has had a positive impact on smoking cessation: smokers’ motivation to quit has increased in the context of the pandemic (22–24). Nearly half (46.50%) of the participants in this study reported that the prevalent use of e-cigarettes is among ex-smokers and that traditional smokers are more likely to express willingness to use e-cigarettes compared to non-traditional smokers. Therefore, smokers’ resolve to smoking cessation will influence their usage of e-cigarettes. It has been noted that both conventional tobacco smokers and e-cigarette users view e-cigarettes as less harmful and addictive. Using e-cigarettes as a method to quit smoking may face challenges due to the possibility of continued e-cigarette use, potentially resulting in dual smoking if complete cessation is not achieved (14, 25, 26). Presently, there is a lack of robust evidence to strongly support the effectiveness of e-cigarettes as a smoking cessation aid. Therefore, it is imperative to ensure adequate and appropriate support for cessation, particularly in light of a potential increase in individuals seeking to quit smoking during and after the COVID-19 pandemic. This can be achieved by expanding the availability of cessation clinics and enhancing counselling and educational resources on smoking cessation provided by healthcare professionals. The majority of participants (96.54%) reported having heard of e-cigarettes, indicating a high level of awareness about e-cigarettes. The results of the questionnaire showed that the medical group have a lower level of willingness to use e-cigarettes relative to the group of non-medical professionals. It is apparent that individuals in the medical field, including both students and experienced doctors, tend to be more aware of the harmful and addictive nature of e-cigarettes, possibly due to their exposure to comprehensive information through medical education and clinical practice (14). However, despite their medical background, the survey results revealed that participants, in general, held misconceptions about e-cigarettes. This highlights the need for further education and clarification on this topic, even within the medical community. For example, participants generally agreed that e-cigarettes contain tar, and the medical group tended to be strongly hold this view, which indicates that the medical group still holds misconceptions about e-cigarettes and lacks knowledge base about them. This phenomenon should be taken seriously considering that numerous e-cigarette users attempt to quit smoking during and after the pandemic (5, 15) and the medical group is the primary provider of smoking cessation counselling and is the most reliable source of information for smokers. Therefore, they should have a more in-depth understanding of smoking cessation to offer better counselling to their patients on quitting smoking. A national survey conducted on medical schools in the United Kingdom concluded that on-campus medical students were not fully trained to offer smoking cessation counselling (12). Similarly, Chinese medical schools lack the appropriate curricula, and students were not adequately prepared to provide cessation counselling (27). Considering medical students are expected to become an important group to provide smoking cessation counselling, further training in smoking cessation counselling is required in medical schools to enhance their competency.

Highly educated individuals possess a more accurate overall understanding of e-cigarettes compared to those with less education. It’s clear that individuals with advanced degrees, which require more years of study, are likely to receive more extensive education, possibly including information about e-cigarettes. However, it’s important to note that participants, including those in the medical group, did not strongly express attitudes towards the costs and policies associated with e-cigarettes. This trend remained consistent across all survey groups, indicating a consistent response regardless of educational background. They did not understand the cost of e-cigarettes and were not clear about the correctness of raising the legal age of e-cigarettes, probably due to their low-level awareness of e-cigarettes. In the context of the pandemic, some smokers opted for e-cigarettes because of their convenience and lower cost (28), and which increased the e-cigarette usage, such as through increasing taxes, may serve as an efficient way to suppress the prevalence of e-cigarette use and promote smoking cessation.

This study has some limitations. Our study employed an online questionnaire, which inherently carries the risk of introducing certain biases. While anonymity was guaranteed and the questionnaire was meticulously designed to mitigate social desirability and measurement biases, the lack of direct interaction might have still impacted participant responses. We recommend future research supplementing online surveys with offline investigation. Despite utilizing a Likert scale and implementing stringent quality control measures, we acknowledge the possibility of residual bias. Specifically, the digital divide may have led to the underrepresentation of certain groups, which could affect the generalizability of our findings. For those working in healthcare whose specialties we did not specifically investigate, respiratory physicians may be more familiar with e-cigarettes. Therefore, whether there are differences between medical specialties requires further investigation. To enhance the survey’s ease, we refrained from assessing the knowledge, attitude, and willingness to use e-cigarettes of medical students before they commenced their studies and graduates before they embarked on their careers. Hence, it is plausible that they might have gained adequate education on this subject earlier, leading to differing perspectives on e-cigarettes. It is our hope that future studies take this into account. Moreover, we did not thoroughly explore the sequence in which individuals were initially introduced to traditional cigarettes and e-cigarettes. Clarifying this aspect may shed light on the true role of e-cigarettes in smoking behavior.

## Conclusion

The present study shows that individuals tend to hold negative attitudes towards the awareness, perceptions, and willingness to use e-cigarettes. Attitudes towards e-cigarettes were not significantly different between medical and non-medical participants. The medical groups were less likely to use e-cigarettes, but misperceptions about e-cigarettes were prevalent. The medical group requires comprehensive training on smoking cessation counselling in the future to prepare for the possible increase in counselling and proper guidance of smokers who are willing to quit after the COVID-19 pandemic. E-cigarettes advertisements are the main sources of information about e-cigarettes to young individuals, and more measures should be taken to restrict e-cigarette advertising and monitor online relevant media discourse. Our study suggests that e-cigarette users may experience increased stress during the COVID-19 pandemic. Further studies are warranted to explore whether targeted stress management strategies could benefit e-cigarette users during times of public health crises.

## Data availability statement

The original contributions presented in the study are included in the article/Supplementary material, further inquiries can be directed to the corresponding author.

## Author contributions

RD: Conceptualization, Writing – original draft, Writing – review & editing, Formal analysis, Investigation, Project administration, Supervision, Validation, Visualization. CY: Conceptualization, Data curation, Investigation, Methodology, Software, Writing – original draft. YY: Data curation, Investigation, Supervision, Writing – review & editing. LL: Investigation, Project administration, Writing – review & editing. XY: Formal analysis, Investigation, Visualization, Writing – review & editing. XiW: Investigation, Validation, Writing – review & editing. JT: Formal analysis, Investigation, Writing – review & editing. YZ: Methodology, Supervision, Writing – review & editing. XuW: Conceptualization, Data curation, Writing – review & editing. HD: Conceptualization, Data curation, Methodology, Project administration, Supervision, Writing – review & editing.

## Footnotes

^1^ http://www.raosoft.com/samplesize.html

## References

[1] Clapp PW Jaspers I. Electronic cigarettes: their constituents and potential links to asthma. Curr Allergy Asthma Rep. (2017) 17:79. doi: 10.1007/s11882-017-0747-5, PMID: 28983782 PMC5995565

[2] Risi S. On the origins of the electronic cigarette: British American Tobacco’s project Ariel (1962–1967). Am J Public Health. (2017) 107:1060–7. doi: 10.2105/AJPH.2017.303806, PMID: 28520481 PMC5463218

[3] Breland A Soule E Lopez A Ramôa C El-Hellani A Eissenberg T. Electronic cigarettes: what are they and what do they do?: electronic cigarettes. Ann N Y Acad Sci. (2017) 1394:5–30. doi: 10.1111/nyas.12977, PMID: 26774031 PMC4947026

[4] Zhu N Zhang D Wang W Li X Yang B Song J. A novel coronavirus from patients with pneumonia in China, 2019. N Engl J Med. (2020) 382:727–33. doi: 10.1056/NEJMoa2001017, PMID: 31978945 PMC7092803

[5] Kalkhoran SM Levy DE Rigotti NA. Smoking and E-cigarette use among U.S. adults during the COVID-19 pandemic. Am J Prev Med. (2022) 62:341–9. doi: 10.1016/j.amepre.2021.08.018, PMID: 34756629 PMC8492610

[6] Bennett M Speer J Taylor N Alexander T. Changes in E-cigarette use among youth and young adults during the COVID-19 pandemic: insights into risk perceptions and reasons for changing use behavior. Nicotine Tob Res. (2022) 25:350–5. doi: 10.1093/ntr/ntac136, PMID: 35639822 PMC9384103

[7] Wang W Lu M Cai Y Feng N. Awareness and use of e-cigarettes among university students in Shanghai, China. Tob Induc Dis. (2020) 18:1–9. doi: 10.18332/tid/125748, PMID: 32994762 PMC7516251

[8] Wang X Zhang X Xu X Gao Y. Perceptions and use of electronic cigarettes among young adults in China. Tob Induc Dis. (2019) 17:17. doi: 10.18332/tid/102788, PMID: 31582928 PMC6751986

[9] Xiao L Parascandola M Wang C Jiang Y. Perception and current use of e-cigarettes among youth in China. Nicotine Tob Res. (2019) 21:1401–7. doi: 10.1093/ntr/nty145, PMID: 30053201 PMC6941706

[10] Wang X Zhang X Xu X Gao Y. Electronic cigarette use and smoking cessation behavior among adolescents in China. Addict Behav. (2018) 82:129–34. doi: 10.1016/j.addbeh.2018.02.029, PMID: 29522934 PMC5893131

[11] Jankowski M Kaleta D Zgliczyński WS Grudziąż-Sękowska J Wrześniewska-Wal I Gujski M. Cigarette and e-cigarette use and smoking cessation practices among physicians in Poland. Int J Environ Res Public Health. (2019) 16:3595. doi: 10.3390/ijerph16193595, PMID: 31557913 PMC6801531

[12] Raupach T Al-Harbi G McNeill A Bobak A McEwen A. Smoking cessation education and training in U.K. medical schools: a National Survey. Nicotine Tob Res. (2015) 17:372–5. doi: 10.1093/ntr/ntu199, PMID: 25257981 PMC5479510

[13] Almutham A Altami M Sharaf F AlAraj A. E-cigarette use among medical students at Qassim University: knowledge, perception, and prevalence. J Family Med Prim Care. (2019) 8:2921–6. doi: 10.4103/jfmpc.jfmpc_567_19, PMID: 31681668 PMC6820393

[14] Al-Sawalha NA Almomani BA Mokhemer E Al-Shatnawi SF Bdeir R. E-cigarettes use among university students in Jordan: perception and related knowledge. PLoS One. (2021) 16:e0262090. doi: 10.1371/journal.pone.0262090, PMID: 34972196 PMC8719738

[15] Kalan ME Ghobadi H Taleb ZB Adham D Cobb CO Ward KD. COVID-19 and beliefs about tobacco use: an online cross-sectional study in Iran. Environ Sci Pollut Res. (2021) 28:40346–54. doi: 10.1007/s11356-020-11038-x, PMID: 33029777 PMC7541093

[16] World Health Organization. Global adult tobacco survey. Available at: https://www.who.int/teams/noncommunicable-diseases/surveillance/systems-tools/global-adult-tobacco-survey (Accessed September 6, 2023).

[17] Gorukanti A Delucchi K Ling P Fisher-Travis R Halpern-Felsher B. Adolescents’ attitudes towards e-cigarette ingredients, safety, addictive properties, social norms, and regulation. Prev Med. (2017) 94:65–71. doi: 10.1016/j.ypmed.2016.10.019, PMID: 27773711 PMC5373091

[18] Tavakol M Dennick R. Making sense of Cronbach’s alpha. Int J Med Educ. (2011) 2:53–5. doi: 10.5116/ijme.4dfb.8dfd, PMID: 28029643 PMC4205511

[19] Chan LL Idris N. Validity and reliability of the instrument using exploratory factor analysis and Cronbach’s alpha. Int J Acad Res Bus Soc Sci. (2017) 7:400–10. doi: 10.6007/IJARBSS/v7-i10/3387

[20] Gentzke AS Wang TW Cornelius M Park-Lee E Ren C Sawdey MD. Tobacco product use and associated factors among middle and high school students — National Youth Tobacco Survey, United States, 2021. MMWR Surveill Summ. (2022) 71:1–29. doi: 10.15585/mmwr.ss7105a1, PMID: 35271557 PMC8923300

[21] Brożek G Jankowski M Zejda J Jarosińska A Idzik A Bańka P. E-smoking among students of medicine—frequency, pattern and motivations. Adv Respir Med. (2017) 85:8–14. doi: 10.5603/ARM.2017.0003, PMID: 28198988

[22] Sharma RH Rodberg D Struik LL. Experiences of nicotine users motivated to quit during the COVID-19 pandemic: a secondary qualitative analysis. BMJ Open. (2023) 13:e070906. doi: 10.1136/bmjopen-2022-070906, PMID: 37369394 PMC10410857

[23] Johnston E Bains M Hunter A Langley T. The impact of the COVID-19 pandemic on smoking, vaping, and smoking cessation Services in the United Kingdom: a qualitative study. Nicotine Tob Res. (2023) 25:339–44. doi: 10.1093/ntr/ntac227, PMID: 36218530 PMC9619632

[24] Hoeppner SS Carlon HA Kahler CW Park ER Darville A Rohsenow DJ. COVID-19 impact on smokers participating in smoking cessation trials: the experience of nondaily smokers participating in a smartphone app study. Telemed Rep. (2020) 2:179–87. doi: 10.1089/tmr.2021.0008 PMC8812286 35720753

[25] Franks AM Hawes WA McCain KR Payakachat N. Electronic cigarette use, knowledge, and perceptions among health professional students. Curr Pharm Teach Learn. (2017) 9:1003–9. doi: 10.1016/j.cptl.2017.07.023, PMID: 29233367

[26] Hajek P Phillips-Waller A Przulj D Pesola F Myers Smith K Bisal N. A randomized trial of E-cigarettes versus nicotine-replacement therapy. N Engl J Med. (2019) 380:629–37. doi: 10.1056/NEJMoa1808779 30699054

[27] Yang C He W Deng R Giri M Dai H. Perceptions and preparedness toward tobacco cessation counseling amongst clinical medical students in Chongqing, Southwest China: a cross-sectional study. Front Public Health. (2022) 10:934782. doi: 10.3389/fpubh.2022.934782, PMID: 35979466 PMC9376593

[28] Chen T Wang L Cheung YTD Wang MP Lam TH Ho SY. Risk perceptions and changes in tobacco use in relation to Coronavirus disease 2019 pandemic: a qualitative study onadolescent tobacco users in Hong Kong. Tob Induc Dis. (2023) 21:92–11. doi: 10.18332/tid/167479, PMID: 37456609 PMC10347963

