# Supplementary material for: A cross-sectional survey of medical and other groups’ awareness, perceptions, and willingness to use e-cigarettes during the COVID-19 pandemic
Source: Front Public Health. 2024 Jan 8;11:1323804. doi: 10.3389/fpubh.2023.1323804 (PMC10800479; doi:10.3389/fpubh.2023.1323804)
Supplement: Supplementary file 1 [file Table_1.docx]

**Screening Methods and Results**

**Screening methods**

1. Frequency analysis method: Check for questions with low response rates in the questionnaire. If the responses are concentrated in one option (>80%), this indicates that the question is difficult to understand for respondents and may consider removing it.

2. Correlation coefficient method: The Pearson correlation coefficient calculated the correlation between each item score and the total score. If the difference is not statistically significant (> 0.05), consider removing it.

3. Cronbach’s alpha method: Calculate the Cronbach's alpha before and after deleting a question, and if the Cronbach's alpha increases, consider deleting it.

4. Discrete degree method: Calculate the coefficient of variation (CV) for each question and consider removing it if CV > 25%

5.Factor analysis method: If the extracted common factor contains only one question, or if a question has a factor loading of <0.400 on a particular common factor, consider removing it

**Screening results**

1. Frequency analysis method: The response rate for all questions was 100%, and percentage of choices without any answer > 80%, so all questions were retained.

2. Correlation coefficient method: The Pearson correlation coefficients between the scores for each question and the total score ranged from 0.203 to 0.671, with all P<0.05, and thus all the topics were retained.

3. Cronbach’s alpha method: After deleting questions 21,22,30, and 31 respectively, the Cronbach's alpha of the remaining scales increased and thus considered for deletion.

4. Discrete degree method: The average scores for each question ranged from 1.61 to 3.46, with standard deviations ranging from 0.948 to 1.616 and coefficients of variation (CVs) of 0.333 to 0.592. All questions were deemed worthy of retention.

5.Factor analysis method: A total of five common factors were extracted from the 18 scale questions, and all questions had factor loadings of >0.400, so all questions were retained. All questions simply loaded larger on only one principal component, thus showing good validity.

After summarizing the six screening methods mentioned above in combination, question 30 and 31 were retained as they were hypothesized factors for this study. Question 21 and 22 were deleted and the number of scale questions were adjusted to 16.

Question 21: E-cigarettes should be regulated or controlled by the government.

Question 22: Raising taxes on e-cigarettes is a good idea.
