# Supplementary material for: A cross-sectional survey of medical and other groups’ awareness, perceptions, and willingness to use e-cigarettes during the COVID-19 pandemic
Source: Front Public Health. 2024 Jan 8;11:1323804. doi: 10.3389/fpubh.2023.1323804 (PMC10800479; doi:10.3389/fpubh.2023.1323804)
Supplement: Supplementary file 2 [file Table_2.docx]

**Questionnaire**

| **1.Demographics and characteristics of participants** | | |
| --- | --- | --- |
| 1 | Age |  |
| 2 | Gender |  |
| 3 | Occupation |  |
| 4 | Education background |  |
| **2.Participants’ smoking habits** | | |
| 5 | Are you smoking now? | Everyday; Yes, but not every day; No |
| 6 | Have you ever smoked every day before? | Yes; No; Unclear |
| 7 | Have you ever smoked before? | Everyday; Yes, but not every day; No |
| 8 | What caused you to start smoking? | The influence of smokers around Feel bored and to kill time Social needs Try something new Release the pressure Other reason |
| **3.participants' perceptions of e-cigarettes** | | |
| 9 | Have you heard of e-cigarettes? | Yes; No |
| 10 | How do you know about e-cigarettes? | Parents, relatives, friends and people around them E-cigarette advertising In TV series and movies Shopping malls and physical stores |
| 11 | In your impression, what kind of people around you use e-cigarettes | People who smoke traditional cigarettes People who quit smoking People who have never smoked traditional cigarettes before smoking e-cigarettes No one uses e-cigarettes |
| 12 | What are e-cigarettes used for | Keep up with fashion trends Relieve anxiety and stress Alleviate smoking addiction and replace traditional cigarettes Social needs Reduce secondhand smoke |
| 13 | In your impression, which age group is the majority of people who smoke e-cigarettes | Over 50, 40 to 49, 30 to 39, 20 to 29, 10 to 19, Under 10 |
| **4.Participants' perception, attitude, and willingness to use e-cigarettes (scale questions)** | | |
| 14 | E-cigarettes are healthier than traditional cigarettes | strongly disagree disagree neutral agree strongly agree |
| 15 | E-cigarettes contain tar | strongly disagree disagree neutral agree strongly agree |
| 16 | E-cigarettes are not addictive | strongly disagree disagree neutral agree strongly agree |
| 17 | E-cigarettes can replace traditional cigarettes and help quit smoking | strongly disagree disagree neutral agree strongly agree |
| 18 | E-cigarettes have no harm from second-hand smoke | strongly disagree disagree neutral agree strongly agree |
| 19 | E-cigarette use is permitted in homes or indoor places such as offices or shopping malls | strongly disagree disagree neutral agree strongly agree |
| 20 | I'd rather use e-cigarettes than traditional cigarettes | strongly disagree disagree neutral agree strongly agree |
| 21 | Raising the legal age for e-cigarettes is a bad thing | strongly disagree disagree neutral agree strongly agree |
| 22 | The price of e-cigarettes is too high | strongly disagree disagree neutral agree strongly agree |
| 23 | Some of my family, friends, and people around me smoke e-cigarettes | strongly disagree disagree neutral agree strongly agree |
| 24 | In the next 12 months, I may use e-cigarettes | strongly disagree disagree neutral agree strongly agree |
| 25 | I have used electronic cigarettes | strongly disagree disagree neutral agree strongly agree |
| 26 | People around me who smoke e-cigarettes will make me want to smoke e-cigarettes | strongly disagree disagree neutral agree strongly agree |
